# Cerebrospinal Fluid Immunoglobulins as Potential Biomarkers of Chikungunya Encephalitis

**DOI:** 10.3201/eid2405.171763

**Published:** 2018-05

**Authors:** Marzia Puccioni-Sohler, Luiz Claudio Farias, Mauro Jorge Cabral-Castro, Mariano G. Zalis, Rosangela S. Kalil, Maria Cecília F. Salgado

**Affiliations:** Universidade Federal do Rio de Janeiro, Rio de Janeiro, Brazil (M. Puccioni-Sohler, L.C. Farias, M.J. Cabral-Castro, M.G. Zalis);; Universidade Federal do Estado do Rio de Janeiro, Rio de Janeiro (M. Puccioni-Sohler, R.S. Kalil, M.C.F. Salgado)

**Keywords:** chikungunya virus, CHIKV, alphavirus, humoral immune response, cerebrospinal fluid, meningitis/encephalitis, viruses, vector-borne infections, immunoglobulins, IgG, IgM

## Abstract

Chikungunya virus causes fever and severe polyarthritis or arthralgia and is associated with neurologic manifestations that are sometimes challenging to diagnose. We demonstrate intrathecal synthesis of chikungunya antibodies in a patient with a history of acute infection complicated by encephalitis. The specificity of the intracerebral immune response supports early chikungunya-associated encephalitis diagnosis.

Chikungunya virus (CHIKV) is an alphavirus transmitted by infected *Aedes* mosquitoes (*Ae.* *aegypti* and *Ae.* *albopictus*) (*1*). Global expansion epidemics have been reported (*1*). The disease is characterized by acute fever, maculopapular rash, headache, and disabling rheumatism (*1*,*2*). Neurologic complications may occur, including encephalopathy, encephalitis, myelitis, and Guillain-Barré syndrome (*3*–*5*). Differentiating CHIKV infection from other arbovirus infections is difficult because of co-occurring conditions and similar manifestations (*3*). Detection of viral RNA and specific antibodies in cerebrospinal fluid (CSF) suggests neurotropic evidence of CHIKV (*2*,*3*), but whether these antibodies are synthesized locally or derived from blood has not been demonstrated (*6*,*7*). We detected intrathecal synthesis of CHIKV IgG by specific antibody index in a case of encephalitis (*6*).

In January 2016, a 69-year-old woman had sudden fever (38°C), intense generalized arthritis, prostration, and cognitive alterations characterized by forgetting and exchanging words. She used analgesics without relief. After 1 week, a maculopapular rash with intense pruritus appeared on her upper limbs. A few days later, her rash and fever abated, but other symptoms continued. She was referred for consultation 3 months after symptom onset. Physical examination revealed bilateral finger and knee arthritis (Technical Appendix Figure). Neurologic examination showed slow thinking, inattention, and mild confusion. She had a history of dengue and Zika virus infections. Results of a routine blood analysis were unremarkable. 

We performed several antibody tests: anti-CHIKV ELISA (IgM and IgG; Euroimmun, Luebeck, Germany); Panbio Dengue IgG Indirect ELISA and Dengue IgM Capture ELISA (Panbio, Brisbane, Queensland, Australia); and immunochromatographic assay (GenBody Zika IgG/IgM; Biotech Business, Chungnam, South Korea). Results for specific IgG and IgM are shown in the Table. CSF analysis demonstrated leukocytes 1 cell/mm^3^; protein 29 mg/dL; glucose 40 mg/dL; blood–CSF barrier function based on albumin quotient 6 × 10^−3^; IgG index 0.43 (reference <0.7); and intrathecal IgG fraction (IgG_IF_) of total IgG found in CSF, <0% (*6*,*7*). In addition, we quantitatively determined the synthesis of specific antibodies by antibody index (AI) (*6*). We used an ELISA test for DENV IgG and ELISA for CHIKV IgG in paired CSF and serum. We calculated AI as the ratio between the specific IgG and total IgG quotient, considering that there was no intrathecal synthesis of total IgG (IgG_IF_ <0%). The sample dilutions for dengue were 1:8 for CSF and 1:4,000 for serum. Sample dilutions for CHIKV were 1:2 for CSF and 1:101 for serum (*6*,*8*). AI was 1.14 for dengue and 7.24 for CHIKV, with AI reference range <1.5 (online Technical Appendix).

**Table:** Results of the detection of IgM and IgG against chikungunya virus, dengue virus, and Zika virus, Brazil, 2016

Target virus	Sample type				
	Serum			Cerebrospinal fluid	
	IgM	IgG		IgM	IgG
Chikungunya	Reactive	Reactive		Reactive	Reactive
Dengue	Not reactive	Reactive		Not reactive	Reactive
Zika	Not reactive	Reactive		Not reactive	Reactive

Brain magnetic resonance imaging showed foci with hyperintense signal in the T2-weighted sequences and fluid attenuation inversion recovery bilaterally in subcortical frontoparietal areas. The patient showed substantial progressive improvement of cognitive alterations and arthralgia after starting antiinflammatory treatment; she took nimesulide for 2 months, followed by prednisolone (20 mg/d) with progressive reduction for another 2 months.

Our results demonstrate the quantitation of intrathecally synthesized CHIKV IgG. We diagnosed CHIKV-associated encephalitis on the basis of fever and altered mental status for >24 hours and positive CHIKV IgM antibodies in serum (*9*). CSF analysis results were unremarkable except for elevated CHIKV AI, the only evidence of brain inflammation. Brain imaging showed unspecific lesions; viral encephalitis may occur without pleocytosis or specific parenchymal abnormalities (*9*).

The identification of specific etiologies of viral encephalitis may be difficult in arbovirus-endemic areas (*3*). Co-infections with Zika virus, CHIKV, and DENV are frequent (*1*,*3*), and neurologic manifestations may also be similar (*4*,*5*). Although this case-patient also had a history of DENV and Zika infection with specific IgG in serum and CSF, we did not detect intrathecal synthesis of DENV antibodies (*6*–*8*) as did Puccioni-Sohler et al. in a previous study (*8*). Our findings show that the quantitation of antibodies synthesized in the brain may be useful in the differential diagnosis of neurologic diseases caused by arboviruses. The detection of specific intrathecal synthesis of antibodies is a known tool for the diagnosis of infections including herpes simplex virus, varicella zoster virus, measles, rubella, neuroborreliosis, and human T-cell leukemia virus type 1 (*6*–*8*).

CHIKV has attracted increasing attention because of its spatial spread and the high number of epidemics. Chikungunya has been associated with debilitating arthropathy for months or years after the initial infection, along with severe neurologic complications such as encephalitis (*4*,*5*,*10*). The detection of the etiologic agent of a central nervous system disease may be difficult, considering that PCR results for CHIKV are positive only during the first days of infection (*1*). In addition, the presence of specific antibodies in CSF may be derived from blood (*6*,*7*). The detection of intrathecal synthesis of CHIKV IgG may be useful as a specific laboratorial brain marker for diagnosis of encephalitis and other neurologic complications associated with CHIKV infection. This result provides evidence of viral neurotropism and can be useful for supporting public health.

Technical Appendix Additional information about chikungunya virus infection in a patient in Brazil.

## References

[1] Burt FJ, Rolph MS, Rulli NE, Mahalingam S, Heise MT. Chikungunya: a re-emerging virus. Lancet. 2012;379:662–71. 10.1016/S0140-6736(11)60281-X 22100854

[2] Kashyap RS, Morey SH, Chandak NH, Purohit HJ, Taori GM, Daginawala HF. Detection of viral antigen, IgM and IgG antibodies in cerebrospinal fluid of Chikungunya patients with neurological complications. Cerebrospinal Fluid Res. 2010;7:12. 10.1186/1743-8454-7-12 20704763 PMC2927496

[3] Acevedo N, Waggoner J, Rodriguez M, Rivera L, Landivar J, Pinsky B, et al. Zika virus, chikungunya virus, and dengue virus in cerebrospinal fluid from adults with neurological manifestations, Guayaquil, Ecuador. Front Microbiol. 2017;8:42. 10.3389/fmicb.2017.00042 28174559 PMC5258761

[4] Ganesan K, Diwan A, Shankar SK, Desai SB, Sainani GS, Katrak SM. Chikungunya encephalomyeloradiculitis: report of 2 cases with neuroimaging and 1 case with autopsy findings. AJNR Am J Neuroradiol. 2008;29:1636–7. 10.3174/ajnr.A1133 18566010 PMC8118764

[5] Scott SSO, Braga-Neto P, Pereira LP, Nóbrega PR, de Assis Aquino Gondim F, Sobreira-Neto MA, et al. Immunoglobulin-responsive chikungunya encephalitis: two case reports. J Neurovirol. 2017;23:625–31. 10.1007/s13365-017-0535-y 28577289

[6] Reiber H, Felgenhauer K. Protein transfer at the blood cerebrospinal fluid barrier and the quantitation of the humoral immune response within the central nervous system. Clin Chim Acta. 1987;163:319–28. 10.1016/0009-8981(87)90250-6 3581475

[7] Sindic CJ, Van Antwerpen MP, Goffette S. The intrathecal humoral immune response: laboratory analysis and clinical relevance. Clin Chem Lab Med. 2001;39:333–40. 10.1515/CCLM.2001.052 11388658

[8] Puccioni-Sohler M, Soares CN, Papaiz-Alvarenga R, Castro MJ, Faria LC, Peralta JM. Neurologic dengue manifestations associated with intrathecal specific immune response. Neurology. 2009;73:1413–7. 10.1212/WNL.0b013e3181bd8258 19858464

[9] Venkatesan A, Tunkel AR, Bloch KC, Lauring AS, Sejvar J, Bitnun A, et al.; International Encephalitis Consortium. Case definitions, diagnostic algorithms, and priorities in encephalitis: consensus statement of the international encephalitis consortium. Clin Infect Dis. 2013;57:1114–28. 10.1093/cid/cit458 23861361 PMC3783060

[10] Vijayan V, Sukumaran S. Chikungunya virus disease: an emerging challenge for the rheumatologist. J Clin Rheumatol. 2016;22:203–11. 10.1097/RHU.0000000000000396 27219309

