# Supplementary material for: Cerebrospinal Fluid Immunoglobulins as Potential Biomarkers of Chikungunya Encephalitis
Source: Emerg Infect Dis. 2018 May;24(5):939–41. doi: 10.3201/eid2405.171763 (PMC5938764; doi:10.3201/eid2405.171763)
Supplement: Technical Appendix — Additional information about chikungunya virus infection in a patient in Brazil. [file 17-1763-Techapp-s1.pdf]

Article DOI: https://doi.org/10.3201/eid2404.171763

Cerebrospinal Fluid Immunoglobulins as Potential Biomarkers of Chikungunya Encephalitis

Technical Appendix

We quantitatively determined the synthesis of specific antibodies in a patient with chikungunya disease by antibody index (AI) as described by Reiber and Felgenhauer (*6*).The antibody index discriminates the pathologic fraction of specific brain-derived CSF immunoglobulin, considering the transfer of blood proteins into CSF. It is based on the analysis of specific antibodies in paired serum and CSF by ELISA technique, and the blood–CSF barrier function (Q_{alb}). We used the Panbio Dengue Indirect IgG ELISA kit (Panbio, Brisbane, Australia) and the Anti-Chikungunya virus (IgG) ELISA kit (EUROIMMUN, Luebeck, Germany). We calculated the antibody index (AI = Q spec/Q IgG) with the ratio between the specific IgG and total IgG, considering that there was no intrathecal synthesis of total IgG (IgG_{IF} <0%). We established a reference curve of 0.05–2.0 absorbance units on the basis of serial dilution of the manufacturer´s positive control. We defined the maximum value of absorbance as 100 arbitrary concentration units. The sample dilutions for dengue virus were 1:8 for CSF and 1:4000 for serum; for CHIKV, 1:2 for CSF and 1:101 for serum (*6*–*8*).

For IgG IF> 0%: $AI = Q_{specific} / Q_{Limit}\ (IgG)$

For IgG IF < 0%: $AI = Q_{specific} / Q_{IgG}$

$Q_{specific}$ = CSF OD x dilution / Serum OD x dilution

Q_{IgG} = CSF total IgG/ Serum total IgG

$Q_{Lim}\ (IgG) = 0.93 \times (Qalb^2 + 6 \times 10^{-6})^{1/2} - 1.7 \times 10^{-3}$

$IgG_{IF} = IgG_{IF} = [Q_{IgG} - Q_{Lim}(IgG)] / Q_{IgG}] \times 100$

Evolution

After 2 months, the patient returned for follow-up; we detected no evidence of neurocognitive disorder from the results of a battery of neuropsychological tests. The assessment tools included a mini mental state examination, Beck depression inventory, Wechsler Memory Scale (logical memory I/ II, visual reproduction, arithmetic, digit span (forward and backward), cube, vocabulary, digit symbol), auditory verbal learning, Rey-Osterrieth Complex figure, trail making test A/B, verbal semantic and phonetic fluency, Stroop Victoria test, and grooved pegboard (dominant and nondominant hands) .

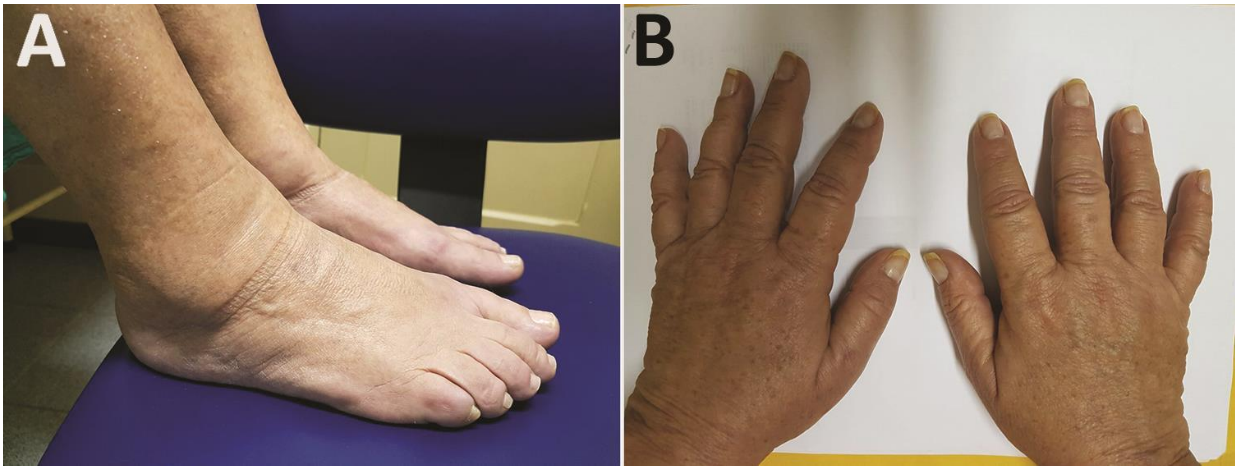

Technical Appendix Figure. Arthritis associated with chikungunya virus infection. Swelling and hyperemia in ankle (A) and fingers (B) of a 69-year-old woman with post-chikungunya tenossinovitis 90 days after acute infection with chikungunya virus, Rio de Janeiro, Brazil.
